# Development of antimalarial drugs and their application in China: a historical review

**DOI:** 10.1186/2049-9957-3-9

**Published:** 2014-03-20

**Authors:** Chang Chen

**Affiliations:** 1 National Institute of Parasitic Diseases, Chinese Center for Disease Control and Prevention, 200025 Shanghai, China

**Keywords:** Antimalarials, Malaria, Treatment, China

## Abstract

This historical review covers antimalarials developed in China, which include artemisinin, artemether, artesunate, and dihydroartemisinin, as well as other synthetic drugs such as piperaquine, pyronaridine, benflumetol (lumefantrine), and naphthoquine. The curative effects of these antimalarials in the treatment of falciparum malaria, including chloroquine-resistant strain, are especially discussed. Following the World Health Organization (WHO) recommended artemisinin-based combination therapy (ACT), different combinations of artemisinin, or its derivative, along with another antimalarial drug were orally used to treat *Plasmodium falciparum* infections. The recrudescence rates were low, gametocyte carriers lessened, and the curative rate increased remarkably. The combination therapy effectively deferred the emergence of drug resistance in the parasite. The regulation “The guidelines and regimens for the use of antimalarial drugs in China” was issued to guide rational application and standardize malaria treatment in the country. As the recommended first-line drug to treat falciparum malaria in the world, ACT was adopted in the regulation. In response to the global initiative of malaria eradication proposed by the UN Millennium Development Goals (MDGs), the Chinese government has set a target to eliminate malaria by 2020.

## Multilingual abstracts

Please see Additional file 1 for translations of the abstract into the six official working languages of the United Nations.

## Review

In China, malaria was not only widespread but also one of the most threatening diseases to people’s health. Historically, traditional Chinese medicinal herbs were used for thousands of years as a folk remedy to treat malaria. Before the 1950s, the antimalarial quinine was imported into the country in great quantities [1].

Quinine was first isolated from the cinchona bark by European scientists in the 1920s. The trees of *Cinchona officinalis* L., Rubiaceae, grew wild in South America and were cultivated in Java, Indonesia. During the Second World War, there was no supply of quinine because Java was occupied by Japanese troops. Because of these circumstances, Chinese scientists tried to isolate an active principle against malaria from the root of the plant *Dichroa febrifuga* L., Saxifragaceae, another traditional herbal medicine, in the early 1940s. The principle was named ‘β-dichroine’ or ‘febrifugine’, and its chemical structure was identified. Two other principles with antimalarial activity were also isolated from the kernel of the plant *Brucea javanica* (L.) M., Simarubaceae, and were named ‘bruceine D’ and ‘bruceine E’, respectively. Their chemical structures were also clarified. However, the investigations did not make further progress into their clinical use [1].

In the early 1940s, chloroquine, an erythrocytic schizonticide of plasmodia, was developed in Western countries. Primaquine and pyrimethamine were synthesized in 1946 and 1951, respectively, and used for radical cure and causal prophylaxis in malaria control.

In 1958, the abovementioned chloroquine, primaquine, and pyrimethamine were also manufactured in China for malaria control. The productive techniques of the drugs were improved in order to reduce the costs to meet the needs of large-scale malaria control [1].

Chloroquine-resistant *Plasmodium falciparum* was found and spread in the early 1960s in Southeast Asia [2]. To improve the research capacities for drug development, animal models were soon introduced or established in the country, including *P. berghei* and the chloroquine-resistant strain of *P. berghei* in mice, and *P. inui* in rhesus monkeys (*Macaca mulatta*). Later, two more experimental models, *P. cynomolgi* and *P. knowlesi* in rhesus monkeys, were established. The new antimalarials were studied and synthesized at a few institutes in the country. Some of the new synthesized compounds were found to have an effect against plasmodia in the blood [1].

During the Vietnamese War in the 1960s, there was an increase of incidence and mortality of chloroquine-resistant falciparum malaria on the border of the Yunnan Province. This situation inspired researchers to actively develop alternative antimalarials that were effective to treat chloroquine-resistant malaria.

To strengthen the research on antimalarials, an extensive cooperation was organized by the Chinese government in 1967 for drug development with participation of relevant institutions and pharmaceutical companies. Systematic researches from chemical syntheses to pharmacotoxicology to clinical trials were carried out. Among the synthetic compounds and principles extracted, a few were clinically proven to be highly effective to treat *P. falciparum* with chloroquine resistance, including artemisinin isolated from a medicinal herb, which, together with its derivatives, aroused a great interest globally at a later stage.

In line with the requirement of malaria control, antimalarial drugs should be rationally used for achieving a curative effect, interruption of transmission, and avoidance or deferment of the development of drug resistance. Therefore, the regulation “The guidelines and regimens for the use of antimalarial drugs in China” was issued and revised in 2009 [3]. Artemisinin-based combination therapy (ACT), proposed by the WHO [4] as a recommended first-line drug to treat falciparum malaria in the world, was adopted in the regulation.

### Artemisinin and its derivatives

**Figure 1:**
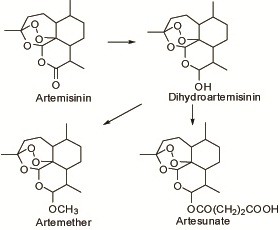
Chemical structures of artemisinins and semi-synthesized derivatives.

In the late 1960s, Chinese scientists investigated active principles against malaria from different medicinal herbs. Various principles showed actions in malaria parasites, and their chemical structures were also determined and identified. Soon after, an important principle was isolated and screened by *P. berghei* in mice. It was a totally new agent: artemisinin. Its derivatives were then semi-synthesized and proven to be active antimalarials (see Figure 1).

#### ***Discovery of artemisinin***

In 1973, an effective principle against malaria was successfully isolated from the aerial part of the plant *Artemisia annua* L., Compositae, and named ‘artemisinin’ (see Figure 1). Interestingly, the other plants in the same genus *Artemisia* did not show any activity in malaria parasites [5]. The plants in *Artemisia*, all called ‘qinghao’, were used for malaria treatment in ancient times. Therefore, artemisinin, isolated from *Artemisia annua*, was also called ‘qinghaosu’ in China.

There is a sesquiterpene lactone with a peroxy linkage in the structure of artemisinin, which is a novel type different from all other antimalarial drugs currently available [6,7].

Experiments demonstrated that artemisinin was an erythrocytic plasmodicide and not active in exoerythrocytic stages of plasmodia. The test in mice infected with chloroquine-resistant *P. berghei* showed a low cross-resistance to chloroquine [8,9]. In an *in vivo* test, artemisinin could be rapidly absorbed, widely distributed, and quickly metabolized, with a half-life of 0.5–0.7 h. The three metabolites found in urine exhibited no action in the parasite [10]. Artemisinin was not soluble in water and oil, and clinically it was applied in the formulas of tablet, water or oil suppositories, or suspension.

Artemisinin showed therapeutic action in the treatment of vivax malaria, however, the curative rate was only about 15% [9]. In the treatment of falciparum malaria, it needed a large dosage, and the recrudescence rate of the parasites was high. Therefore, the drug is not used alone in malaria treatment but in combination with another antimalarial drug.

The poor solubility and low curative rate of artemisinin stimulated the researchers to transform its chemical structure and synthesize water/oil-soluble derivatives [11]. The effect of those derivatives was better than that of the parent artemisinin. Among them, artemether, artesunate, and dihydroartemisinin were developed successively to form the novel antimalarial drugs, artemisinins (see Figure 1).

#### ***Quality of artemisinins***

Artemisinins showed similar qualities to each other (see Figure 1). They were effective against erythrocytic stages of plasmodia and possessed low cross-resistance to chloroquine in the chloroquine-resistant strain of *P. berghei* in mice [8,9]. The antimalarial action of artemisinins occurred in the membrane structure; initial action on the membrane of food vacuoles, mitochondria membrane, etc., and autophagic vacuoles were formed to kill parasites [8].

The experiments of subacute toxicity in animals showed that artemisinins could dose-dependently increase brainstem neurotoxicity [12]; the audit sensory was easily affected. The toxicity of artemether was high, medium in dihydroartemisinin, and low in artesunate and artemisinin. Clinically, however, the toxicity was not reported. Other studies revealed that the toxic effect of artemisinins was largely manifested in the haematopoietic cells of the bone marrow, especially those of the erythroid series. In addition, the toxic effect on the cardiac muscle and liver was also found following the administration of artemisinins into animals [13,14], but no similar clinical toxicity was observed in humans. Studies in mice and rats indicated that artemisinins showed an apparent embryotoxicity. Both dead fetus and absorption of fetus were observed in gestational mice and rats. No teratogenicity was found, but artesunate administered even with a small dose exhibited the above fetal toxicities, with teratogenesis of the umbilical hernia in fetus and dead fetus [15]. Therefore, pregnant women were advised to be cautious with artemisinins and the drugs were contraindicated to women in their first trimester of pregnancy.

#### ***Artemether***

Artemether (see Figure 1) was obtained through semi-synthesis from artemisinin in 1976 [11]. It was still insoluble in water, but soluble in oil, therefore it could be formulated as a tablet or groundnut oil injection for application in the clinic. It was sensitive to light and heat, and slowly decomposed which meant its content dropped if stored for a longer time [16,17]. The elimination half-life of the drug was 0.90 ± 0.37 h in the body [18].

The cases with vivax malaria were intramuscularly treated by a total dosage of 640 mg of artemether. The fever subsided and the asexual parasites were cleared in malaria cases, but the recrudescence rate was high [19]. Generally, artemether was not used to treat vivax malaria.

The cases of falciparum malaria were intramuscularly treated with a total dosage of 240 mg or 640 mg of artemether in three or five days. The average fever subsidence time was within two days and the average parasite clearance time was within three days. The recrudescence rate for one month was 10.5–20% [20,21]. It did not show gametocidal activity [20]. If the total dosage was 600 mg by oral ingestion in the course of five days, the recrudescence rate of treated falciparum malaria cases was 14% [22].

Finally, an intramuscular injection regimen with a total dosage of 640 mg of artemether, divided into eight doses (double doses on the first day and one dose each for the next six days) was recommended for the treatment of falciparum malaria [3].

#### ***Artesunate***

Artesunate was developed by semi-synthesis from artemisinin [23] (see Figure 1). It was also poorly soluble in water, however, sodium artesunate was soluble in water and injectable. For clinical use, 5% sodium bicarbonate injection was added to 60 mg of artesunate, which was then diluted with 5% glucose to obtain a 6 ml solution of sodium artesunate. It had to be immediately administered by intravenous or intramuscular injection, and not be used by intravenous drip because the solution was unstable and soon decomposed. Its elimination half-life in the body was 0.6–0.8 h [24].

One hundred and fifty-nine patients with falciparum malaria received a total dosage of 400 mg of artesunate administered by intravenous or intramuscular injection in the course of three days. The mean fever subsidence and parasite clearance times were 26.1 h and 28.5 h, respectively. The recrudescence rate was as high as 52.4% [19]. The total dosage was increased to 600 mg by oral ingestion in the course of five days in the treatment of falciparum malaria; the recrudescence rate was still high (26.7%) [25].

One hundred and six patients with cerebral malaria received a regimen of 420 mg of artesunate by injection over six days. Ninety-eight patients were cured and eight died [26].

Consequently, a total dosage increased to 480 mg by injection over seven days (60 mg/dose; two doses on the first day, one dose each for the next six days) was recommended in the treatment of falciparum malaria [3].

#### ***Dihydroartemisinin***

The semi-synthetic dihydroartemisinin was a middle compound in synthesis (see Figure 1) and an active metabolite from artemether and artesunate in the body. It was an erythrocytic schizonticide with an elimination half-life of 2.6 h in the body [27].

A total dosage of 480 mg of dihydroartemisinin was divided into eight doses by oral ingestion (two doses on the first day and one dose each for the next six consecutive days). The regimen was given to 64 patients with falciparum malaria. The mean times of fever subsidence and parasite clearance were two and three days, respectively. The recrudescence rate was about 4% followed-up for 30 days or 35 days. Gametocytes were still present 14 days after treatment [28,29]. The regimen was used to treat patients with vivax malaria. The mean times of fever subsidence and parasite clearance were two days. The recrudescence rate was as high as 30.6% [28]. Currently, it is not used to treat vivax malaria.

#### ***Side effects of artemisinins***

Clinically, the side effects of artemisinins were similar. In general, they were well tolerated. Some patients complained of dizziness, headache, nausea, vomiting, abdominal pain, diarrhea, and palpitation, etc. A few patients had rashes which disappeared after treatment with suitable medicine. Some patients had reticulopenia, leucopenia, and increased SGPT, and urea nitrogen, sinus bradycardia, arrhythmia or premature ventricular beat. Usually the side effects disappeared in one to three days or seven days after treatment.

### Synthetic new antimalarial drugs

**Figure 2:**
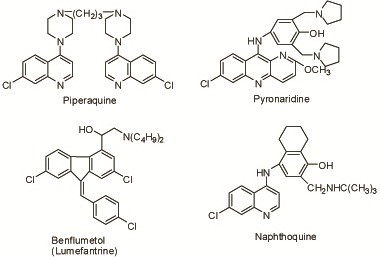
Chemical structures of synthetic antimalarial drugs.

Chemical syntheses have been another important way for developing new antimalarials. Among numerous chemicals, four compounds (see Figure 2) showed remarkable action in malarial parasites and were developed as new antimalarials in the country between the 1960s and the 1980s (see Table 1).

**Table 1:** Artemisinins and other antimalarials

***Drug***	***Date of discovery or creation***	***Initial action in parasite***	Cross- resistance to CQ*	***Half-life (h)***	***Route of administration for treatment***	***Reference***
Artemisinin	1973	Membrane system, on membrane of food vacuoles etc.	Low	0.5–0.7	po	[5,8-10]
Artemether	1976		Ditto	0.90 ± 0.37	po, im	[8,11,18]
Artesunate	1977		Ditto	0.6–0.8	po, im, iv	[23,24]
Dihydroartemisinin	1976		Ditto	2.6	po	[11,27]
Piperaquine	1965	Food vacuoles	Possession	216	po	[30-32]
Pyronaridine	1970	1. Food vacuoles	Without	65,251**	po, im, iv drip	[38-45]
		2. Pellicular complexes				
Benflumetol	1976	***	***	47.4	po	[51,52]
Naphthoquine	1981	Membrane system	Possession	41–57	po	[56]

*The tests were against *P. berghei* in mice. CQ: chloroquine.

**By different methods.

***The initial action and cross-resistance of the drug were reported to be similar to those of mefloquine.

#### ***Piperaquine***

Originally developed in France in 1963, piperaquine (see Figure 2), identical to 13228RP, with long-effective action against malaria, was synthesized by imitation in China in 1965. It was active in the erythrocytic stage of malarial parasites and had a long-term effect on the suppressive prophylaxis of malaria. The drug showed no action in the exoerythrocytic stage of the parasites, and was proved to have cross-resistance to chloroquine [30]. It was retained in the body for a long time after oral administration with an elimination half-life of about nine days. A large amount of the drug was stored in the liver, kidney, and other tissues after oral use in mice, and the release was delayed [31]. Its metabolite in the body was not found. Its killing effect on the asexual forms was due to the interference to the structure of food vacuoles of *P. berghei* in mice [32].

Field trials of the suppressive prophylaxis were carried out in the transmission season in Hainan. Five thousand, seven hundred and eighty-one person-times orally received a single dose of 600 mg of piperaquine monthly from June to September in 1973; the monthly incidence decreased correspondingly from 3.2% to 1.4%. The incidence of the non-medicated control group with 264 persons in the same residential area was 8.0% in June and 10.3% in September. With the parasite carriers as subjects, 376 received the above regimen from June to September in 1974. The rate of parasite carriers reduced from 33.8% before administration to 10.6% after the last administration. Among the subjects, 57.6–59% displayed recrudescence or illness 21–30 days after dosing, indicating that the actual period of suppressive prophylaxis afforded by the drug was about 20 days [33].

A total oral dosage of 1.5 g of piperaquine was given to cases with falciparum malaria. The dosage was divided into five doses (two doses the first time, one dose after a 6 h interval, and two the next day). The mean fever subsidence time was about two days and the mean parasite clearance time was about three days. The recrudescence rate was 14.9% or 18% [34,35], followed-up for 30 days. The cases with gametocytes were 74.5% after treatment [34].

The side effects noted were headaches, dizziness, listlessness, somnolence, vomiting, abdominal pain, diarrhea, facial tingling, and reduction of reticulocytes. They were mild and recoverable after treatment. A few cases showed asthma, stuffiness in the chest or dyspnea, palpitations, or I° A-V block, which disappeared soon after a proper rest. A few women complained of irregular menstruation which normalized after two months. Piperaquine was contraindicated to the patients with severe acute hepatism, nephropathy, and heart disease, and pregnant women were advised to use with caution.

In the late 1980s in China, piperaquine was used as a prophylactic and therapeutic agent in malaria control for 10 years. The resistance rate of *P. falciparum* to the drug was 72.9–96.45% [36], with a wide distribution in the endemic area in southern China. In 1993, 51 cases of falciparum malaria received piperaquine with the routine treatment. Only 17 cases were sensitive to the drug and the average time of parasite clearance was 105 ± 17 h. The parasites in 34 cases were not cleared seven days after treatment. Respectively, 10, 11, and 13 cases showed RI-RIII resistance [37].

Piperaquine is presently used only for suppressive prophylaxis of vivax malaria in specific populations, i.e. mobile populations such as migrant workers, personnel making crossings at the border, travellers, etc. This drug can accumulate in the liver, and should not be given for longer than four consecutive months [3].

#### ***Pyronaridine***

Pyronaridine (see Figure 2), a new erythrocytic schizonticide, was synthesized in China in 1970. No cross-resistance to chloroquine was found [38,39]. Series of toxicological experiments demonstrated that the toxicities of pyronaridine were less significant than those of the chloroquine control group.

Pyronaridine exerted its lethal action primarily in both food vacuoles and pellicular complexes of the parasites in rodent and primate models, while chloroquine exhibited its primary action in the food vacuoles only and there was no significant action in the pellicular complexes. Interestingly, the effect on the pellicular complexes persisted in the chloroquine-resistant strain of *P. berghei* in mice when pyronaridine was given, while the food vacuoles showed no significant change. Chloroquine and mefloquine showed no significant changes in both food vacuoles and pellicular complexes against the resistant isolates. This observation might provide a clue of the effectiveness of pyronaridine against chloroquine, as well as multidrug-resistant isolates of *P. falciparum* and no cross-resistance between pyronaridine and chloroquine [40-43].

The absorption of pyronaridine was rapid and widely distributed in the body. Its elimination half-life in humans was 65 h [44] or 251 h [45]. The difference in the estimation of the half-life might be explained by the shorter observation period used in the study of the shorter half-life and by the difference in the detection limits of the analytical methods.

Pyronaridine could dose-dependently increase the rate of fetal resorption. It possessed embryotoxicity, which was still lower than that of the known positive control drug, dexon (*P* < 0.001). No teratogenesis was noted [46]. Pregnant women were advised to use the drug with caution.

The total oral dosage was 1.2 g or 1.6 g divided into four doses (two doses on the first day at 4–6 h intervals, one dose each for the next two days). The total dosage by intramuscular injection or intravenous drip was 100–300 mg divided into two doses given at 4–8 h intervals. The regimens of pyronaridine in the treatment of malaria were efficient and safe. Generally, the mean fever subsidence time of the patients infected by *P. vivax* or *P. falciparum*, or the chloroquine-resistant strain of *P. falciparum*, was one to two days and the mean parasite clearance time was one to three days. The mean parasite clearance time by parenteral administration was shorter than that by oral administration, and the parasite clearance time for vivax malaria was shorter than that for falciparum malaria [47]. The recrudescence rate of vivax and falciparum malaria was about 10%, followed-up for 28 days or a month after treatment.

Over 100 cases of cerebral malaria and other severely complicated malaria were successfully cured with emergency treatment with pyronaridine.

More than 10 pregnant patients during their mid or late trimester were also cured with pyronaridine without any adverse effects.

In general, pyronaridine was well tolerated by patients. Some patients complained of discomfort in the stomach or epigastrium, dizziness, nausea, headache, abdominal pain, and slight diarrhea. A few patients experienced vomiting, palpitation, and allergic skin rashes which recovered in two days after suitable medicine was taken.

Pyronaridine was used orally to treat falciparum or multidrug-resistant falciparum malaria in Africa, specifically in children, and *P. ovale* and *P. malariae* infections. The patients were all cured with no recrudescence. The side effects were minor and transient [48-50].

#### ***Benflumetol (lumefantrine)***

Benflumetol or lumefantrine (see Figure 2) was synthesized in China in 1976 [51]. It was insoluble in water or oils, but soluble in unsaturated fatty acids such as linoleic acid, and therefore formulated as pills of linoleic acid for oral administration.

The drug was effective in the erythrocytic stage of malaria parasites, was less toxic, absorbed slowly, and distributed rapidly in the body. The elimination half-life in humans was 47.4 h. The cross-resistance of benflumetol to chloroquine was similar to that of mefloquine [52]. Subacute toxicity studies indicated that atrophic degeneration of the kidney glomerulus and liver degeneration were observed in rats and dogs, but these changes were reversible on withdrawal. Slight hematopoietic depression and an increase of leukocytes were also observed in rats; these changes were also reversible. Benflumetol was not mutagenic, teratogenic, and did not affect reproduction in rats [53].

In Hainan, a total oral dosage of 3.6 g of benflumetol divided into 18 or 12 doses (three doses daily at 8 h intervals for six or four consecutive days) was used to treat falciparum malaria. The mean times of fever subsidence and parasite clearance were three days and two days, respectively, with a recrudescence rate of 11.5%, and most patients carried gametocytes for a long time after medication [54]. A total dosage of 2.4 g of benflumetol over a four-day course was used. The mean times of fever subsidence and parasite clearance were 29.4 ± 24.9 h and 54.7 ± 17.4 h, respectively. The recrudescence rate was 4.1% [55].

In Yunnan, 61 cases with falciparum malaria were treated by an oral total dosage of 2.0 g divided into five doses (two doses on the first day, one dose daily for the next three days). The average times of fever subsidence and parasite clearance were within 60 h. Two days after treatment, four out of 61 cases showed a high fever (39°–40.4°C) and with the rate of asexual forms being 48–261%. The four cases failed treatment. The recrudescence rate was 9.8% (five out of 51 cases), followed-up for 28 days. The occurrence rate of gametocytes was 3.9% 28 days after treatment [22]. The failed four cases might be induced by the lower sensibility of *P. falciparum* to the drug or by a lower dosage of the drug. The side effects of the drug included diarrhea, abdominal pain, vomiting, dizziness, etc., which could disappear after a nap.

#### ***Naphthoquine***

Naphthoquine (see Figure 2), synthesized in China in 1981, was effective in the erythrocytic stage of malaria parasites and showed cross-resistance to chloroquine in the test of mice infected with *P. berghei*[56]. The concentration of naphthoquine in the red blood cells was higher than that in the blood plasma, with an elimination half-life of 41–47 h.

In Hainan, 101 patients with falciparum malaria received a total oral dosage of 1.0 g naphthoquine given in dosages of 600 mg and 400 mg on the first and second day, respectively. The average times of fever subsidence and parasite clearance were 50.9 ± 38.8 h and 58.0 ± 21.2 h, respectively. Parasites in three patients were not cleared seven days after treatment until day 14, 21, and 29, respectively. The recrudescence rate was low [57].

No significant side effects; uncomfortable epigastrium, abdominal distension, and, occasionally, urine in the blood could occur [3].

### A combination therapy of artemisinins with another antimalarial drug

The results of monitoring the sensitivity of *P. falciparum* to antimalarials in the field demonstrated that the sensitivity of *P. falciparum* to artemisinins was declining [58], and the effect of artemisinins was likewise decreasing in the treatment of falciparum malaria in which the resistant cases possibly emerged [59-64]. In order to defer the development of resistance to artemisinins, the WHO strongly recommended that artemisinin-based combination therapy (ACT) is applied to treat falciparum malaria. Therefore, artemisinin and its derivatives were used in combination with another synthetic antimalarial drug to treat falciparum malaria.

#### Compound tablet of artemisinin and piperaquine (co-artemisinin)

Artemisinin (62.5 mg) and piperaquine (375 mg), in a fixed-ratio of 1:6, was mixed to form one compound tablet of artemisinin and piperaquine, or co-artemisinin. A total oral dosage of the compound tablet was four tablets divided into two doses (one dose daily for two consecutive days) to treat falciparum malaria [3].

#### ***Compound tablet of dihydroartemisinin and piperaquine***

Dihydroartemisinin (40 mg) and piperaquine phosphate (320 mg), in a fixed-ratio of 1:8, was formulated to obtain one compound tablet of dihydroartemisinin and piperaquine. An oral total dosage was four tablets (one tablet each at 0 h, 6–8 h, 24 h, and 32 h, respectively). Sixty-two cases of falciparum malaria were treated in Yunnan, with average times for fever subsidence and parasite clearance of 34.99 ± 16.51 h and 33.14 ± 11.91 h, respectively. The negative rate of gametocytes was 95.5%. No recrudescence was found 28 days after follow-up [65]. Similar results with a low recrudescence rate were obtained in the treatment of falciparum and drug-resistant falciparum malaria in Cambodia, Myanmar, and other areas [66-71].

#### ***Compound tablet of artemether and benflumetol (co-artemether)***

One compound tablet of artemether and benflumetol (lumefantrine), or co-artemether, was formed by combining artemether (20 mg) with benflumetol (120 mg), in a fixed-ratio of 1:6. Synergism was shown between the two drugs. An oral dosage was 16 tablets divided into four doses (one dose each at 0 h, 8 h, 24 h, and 48 h, respectively). One hundred and sixty-seven cases with falciparum malaria were treated in 2005. The mean fever subsidence and parasite clearance times were 20.4 ± 8.4 h and 37.9 ± 7.9 h, respectively. The recrudescence rate was 5.9%, followed-up for 28 days [72]. In previous field trials in the treatment of falciparum malaria, similar results with mild side effects and 2.1% of gametocytes carriers were obtained [22,55]. This compound tablet could overcome the high recrudescence rate by using artemether alone or by slow action of benflumetol alone in the treatment of falciparum malaria.

#### ***Compound tablet of artemisinin and naphthoquine (co-naphthoquine)***

Artemisinin (125 mg) and naphthoquine (50 mg), in a fixed-ratio of 2.5:1, was mixed to form one compound tablet of artemisinin and naphthoquine, or co-naphthoquine.

A total oral dosage of eight tablets was given as a single dose to 100 patients with falciparum malaria in Hainan. The average fever subsidence and parasite clearance times were 17.5 ± 12.3 h and 30.0 ± 8.8 h, respectively. The recrudescence rate was 3%. The adverse reactions included headache, nausea, weakness, and loss of appetite. These were recoverable two days after withdrawal [73]. Naphthoquine could cause urine in the blood; the compound tablet should stop being taken immediately if that occurs [3].

#### ***Artemisinin derivative combined with pyronaridine***

A combination therapy of dihydroartemisinin (300 mg) plus pyronaridine (800 mg) divided into four doses (two doses on the first day at 4–6 h intervals, one dose each for the next two days) was orally administered. The two separate drugs were simultaneously taken. Thirty-two patients with multidrug-resistant falciparum malaria were treated. A double blind clinical trial was performed with routine dosage of dihydroartemisinin or pyronaridine alone as control groups in Hainan in 2001. The mean fever subsidence time of the combination therapy group was 35.7 ± 24.7 h, and asexual forms clearance time was 23.8 ± 10.1 h. In the group taking dihydroartemisinin alone, the recrudescence of parasites was found in one case with a temperature of 39.6°C on day 21, who failed the treatment, and in four cases *P. vivax* appeared between day 21 and 28 after treatment. No recrudescence was found in the group taking pyronaridine alone and the combination group, followed-up 28 days. The gametocyte carriers occupied 16.7% in the dihydroartemisinin group, 60.9% in pyronaridine group, and 20.0% in the combination group, significantly lower than the group of pyronaridine alone (*P* < 0.01). No abnormality was found in blood, urine, and ECG examinations before or after treatment. A few patients complained of headaches and dizziness, but recovered in one to two days [74]. It was considered that the combination therapy was the ideal medication in the treatment of multidrug-resistant falciparum malaria [74].

The above regimen of dihydroartemisinin plus pyronaridine was applied in the state of Eritrea, Africa in 2000. Thirteen cases with chloroquine-resistant falciparum malaria received the regimen, and similar results were obtained. Gametocyte carriers were found in 18% of the cases on day seven after treatment [75].

A total oral dosage of artemether or artesunate (300 mg) or dihydroartemisinin (200 mg) plus pyronaridine (800 mg), in a course of two days and given as two separate drugs at the same time, was taken for the treatment of falciparum malaria. No recrudescent rate, and the gametocytes disappeared on day 20 after treatment. Less adverse effects were recorded [76,77].

The Shin Poong Pharmaceutical Co. and WHO/TDR/PRD, as partners, have developed the pyronaridine-artesunate project for the treatment of uncomplicated malaria [78].

#### ***Artesunate combined with amodiaquine***

An oral total dosage of artesunate (600 mg) plus amodiaquine (1.8 g) was divided into three doses, one each for three consecutive days. The two separate drugs were taken simultaneously for the treatment of *P. falciparum* infections. As amodiaquine could lead to agranulocytosis and liver damage [79], it was not used in malaria prophylaxis, and rarely used in malaria treatment in China. If there is an adverse reaction, this combination therapy should be discontinued immediately [3].

Amodiaquine possessed cross-resistance to chloroquine. In 1996, an *in vitro* test revealed that the resistance rate of *P. falciparum* to amodiaquine was 85.3–100% [36], which was similar to, or higher than, chloroquine in the Yunnan Province. The combination therapy with amodiaquine should be closely monitored if used.

### Application of antimalarial drugs in the control program

In view of the malaria situation and the drug-resistance developed in *P. falciparum* in the country, and based on the experience from other countries, ACT and “The guidelines and regimens for the use of antimalarial drugs in China” (issued in 2000 and revised in 2009 [3]) are the guidelines which set out how to treat and control malaria.

For the treatment of vivax malaria, a predominant species and still sensitive to chloroquine, an oral dosage of 1.2 g of chloroquine in a three-day course, plus a dosage of 180 mg of primaquine in an eight-day course is recommended as routine therapy of radical cure. The dosage of 180 mg of primaquine in an eight-day course is designed as an anti-relapse treatment of vivax malaria.

As for prophylaxis in specific populations, i.e. mobile populations such as the ones mentioned previously, a single dose of 600 mg of piperaquine orally administered once monthly is recommended. A single dose of 300 mg of chloroquine, once every 7–10 days, is also recommended for prophylaxis.

These drugs are evidently effective and affordable for vivax malaria control. There are large numbers of vivax malaria cases in the country and therefore, the drugs for treatment of vivax malaria are considered as the first-line antimalarials in China.

Because of the persistent efforts in the past decades to control malaria, the number of indigenous falciparum malaria cases has considerably decreased and accounts for about 10% of the total malaria cases, mostly evident in the border area of Yunnan and in the forest hill area of Hainan. At the end of 2012, the percentage decreased to 6% [80], showing that indigenous cases of falciparum malaria are in the minority. The drugs in the combination therapy to treat falciparum malaria, as mentioned above, are considered as second-line antimalarials in China, but ACT is a recommended first-line drug to treat falciparum malaria in the world.

For the emergency treatment of cerebral or other complicated, severe malaria cases, artemether, artesunate, or pyronaridine can be the drugs of choice, administered by intramuscular or intravenous injections, or intravenous drip. These drugs are considered as the third-line antimalarials.

## Conclusion

Drug development is one of the most important components in malaria control due to the critical situation of drug resistance of *P. falciparum*. Chinese scientists have made great contributions to the world by developing new drugs, including artemisinins and a few others. These progresses have made it possible for artemisinin-based combination therapy (ACT) to not only effectively cure numerous cases but also defer the emergence of resistance of *P. falciparum* to the antimalarials.

During treatment, the results were sometimes not the same for the same drug or the same drug combination therapy. Besides the different fields, the different results can be explained by the different dosages, courses of treatment, and the numbers of cases which followed-up. Generally, artemisinins are rapidly active against plasmodia, have a long course, and have a high recrudescence rate. After treatment with artemisinins, the gametocyte carriers are less significant than those with piperaquine, benflumetol, and pyronaridine. The actions against malaria parasites are slow when benflumetol and naphthoquine are used alone, and a large dosage of benflumetol is needed. It has been suggested that naphthoquine should be used in combination with a rapid active antimalarial [57]. By using drug combination therapy to treat falciparum malaria, the recrudescence rate was low, the number of gametocyte carriers lessened, and the curative rate increased remarkably. If the gametocytes still persisted after treatment, a suitable dose of primaquine will be given to eliminate the remaining gametocytes for interrupting the spread of *P. falciparum*.

With the varying results, the suitable treatment regimens for the drugs and drug combination therapies should be provided with regulations. Therefore, the regulation “The guidelines and regimens for the use of antimalarial drugs in China” was issued by the health authority. As a recommended first-line drug to treat falciparum malaria in the world, ACT has been adopted in the regulation. It provides the norms for drug selection and standardized treatment of malaria cases. It is also a valid measure to avoid or defer the development of drug resistance of *P. falciparum*.

As a response to the initiative of global eradication of malaria proposed by the UN Millennium Development Goals, and on the basis of the great successes achieved in malaria control in the past decades, since 2010, the Chinese government has launched a program to eliminate malaria in most regions by 2015, and in the whole country by 2020 [81].

## Competing interests

The author declares that he has no competing interests.

## Supplementary Material

Additional file 1 Multilingual abstracts in the six official working languages of the United Nations. Click here for file
