# Supplementary material for: Development of antimalarial drugs and their application in China: a historical review
Source: Infect Dis Poverty. 2014 Mar 20;3:9. doi: 10.1186/2049-9957-3-9 (PMC4021599; doi:10.1186/2049-9957-3-9)
Supplement: Additional file 1 — Multilingual abstracts in the six official working languages of the United Nations. [file 2049-9957-3-9-S1.pdf]

Translation of the abstract into the six official working languages of the United Nations

تطوير عقاقير مضادة للملا ريا وتطبيقاتها في الصين: استعراض تاريخي

شانغ شن

ملخّص

يغطي هذاالإستعراض التاريخي العقاقير المضادة للملاريا التي تم تطويرها في الصين وهي تضم الأرتيميسينين والأرتيميثير والأرتسونات و dihydroartemisinin إضافة إلى عقاقير اصطناعية أخرى مثل بايبراكين و بيروناريدين وبنفلوميتول(لومفنترين) ونفتوكين. إن الآثار العلاجية لهذه العقاقير المضادة للملاريا في معالجة الملا ريا المنجلية بما فيذلك المستفردات المقاومة للكلوروكين قد تمت مناقشتها بصفة خاصة.

تماشيا مع منظمة الصحة العالمية (WHO) التي أوصت بمجموعات من العقاقير المركبة المستندة على الأرتيميسينين(ACT) فقد تم استخدام مجموعات مختلفة من الأرتيميسين أو مشتقاته عن طريق الفم من أجل معالجة العدوى الناتجة عن المتصورة المنجلية. كانت معدلات الإنتكاس منخفضة وحاملات عرسيات الملاريا أقل مع ارتفاع معدل الشفاء بصفة ملحوظة. ساهم العلاج المركب بصفة فعالة من تأجيل ظهور مقاومة الطفيلية للأدوية. إن صدور لائحة" المبادئ التوجيهية ونظم استخدام الأدوية المضادة للملا ريا في الصين" جاءت لتوجيه التطبيق العقلاني وتوحيد علاج الملاريا في البلاد". وحسب العلاج المنصوح به عند المرحلة الأولى للعلاج الدوائي من الملا ريا المنجلية في العالم، فقد تم اعتماد العقاقير المركبة المستندة على الأرتيميسينين (ACT) في اللائحة. وتجاوبا مع المبادرة العالمية للقضاء على الملاريا والمقترح في إطار الأهداف الألفية للتنمية للأمم المتحدة(MDGs) فقد حددت الحكومة الصينية هدف القضاء على الملاريا في غضون سنة 2020.

Translated from English version into Arabic by Malika2012, through

TRANSLATORS
WITHOUT BORDERS

中国抗疟药的发展及其应用：历史回顾

陈昌

摘要：

本文对中国抗疟药的发展进行历史回顾，包括青蒿素、蒿甲醚、青蒿琥酯和双氢青蒿素以及合成的抗疟药哌喹、咯萘啶、本芴醇(或称 Lumefantrine)和萘酚喹。尤其对这些药物治疗恶性疟和抗氯喹恶性疟进行了讨论。随着世界卫生组织(WHO)倡导的应以青蒿素类抗疟药为基础的联合疗法(ACT)治疗恶性疟，从而以青蒿素及其衍生物分别与另一种其他抗疟药组成不同的复方或联合应用，口服治疗恶性疟，取得治愈率高、原虫复燃率和治后配子体携带率低的效果；且发挥延缓恶性疟原虫对治疗的药物产生抗药性的作用。为了指导合理使用抗疟药和规范治疗用药，在国内印发了“抗疟药使用原则和用药方案（修订稿）”的准则。ACT 作为全球治疗恶性疟的一线药物，已被上述准则所采用。为响应联合国千年发展目标(MDGs)提出的在全球根除疟疾的倡议，中国政府决定到 2020 年全国实现消除疟疾的目标。

Translated from English version into Chinese by Chen Chang

Développement de médicaments antipaludéens et leur mise en application en Chine : Une analyse historique

Chang Chen

Résumé

Cette analyse historique passe en revue les traitements antipaludéens développés en Chine, qui incluent l'artémisinine, l'artéméther, l'artésunate et la dihydroartémisinine, ainsi que d'autres médicaments synthétiques tels que la pipéraquine, la pyronaridine, le benflumétol (luméfantrine), et la naphtoquine. Les effets curatifs de ces antipaludéens dans le traitement du paludisme à *Plasmodium falciparum*, y compris les isolats résistants à la chloroquine, sont débattus en particulier. Suite à la recommandation de l'Organisation Mondiale de la Santé (OMS) en faveur d'une association thérapeutique à base d'artémisinine (ACT), différentes associations d'artémisine, ou de ses dérivés, avec d'autres médicaments antipaludéens ont été utilisées par voie orale pour traiter les infections à *Plasmodium falciparum*. De faibles taux de recrudescence ont été observés, tout comme une diminution des porteurs de gamétocytes, et une remarquable augmentation des taux de guérison. Le traitement en association a retardé de façon efficace l'émergence de résistance pharmacologique du parasite. La réglementation « Lignes directrices et posologies pour l'utilisation des médicaments antipaludéen en Chine » a été émise pour aiguiller la mise en application et la standardisation du traitement antipaludéen dans le pays. Les ACT, traitement de première intention du paludisme à *Plasmodium falciparum*, ont été adoptés dans la réglementation. En réponse à l'initiative mondiale d'éradication du paludisme proposée dans les Objectifs du Millénaire pour le Développement (OMD) des Nations Unies, le gouvernement chinois s'est fixé pour but d'éliminer le paludisme d'ici à 2020.

Translated from English version into French by NathC, through

TRANSLATORS
WITHOUT BORDERS

Развитие и применение в Китае противомалярийных лекарственных препаратов: исторический обзор

Чанг Чен (Chang Chen)

Фрагмент

Этот исторический обзор охватывает противомалярийнее средства, разработанные в Китае. К ним относятся артемизинин, артеметер, артесунат и дигидроартемизинин, а также другие синтетические препараты, такие как пиперакин, пиронаридин, бенфлуметол (лумефантрин) и нафтокин. Особо обсуждается лечебный эффект этих противомалярийных препаратов, направленный на лечение малярии, в том числе, хронической-стойкой малярии. Всемирной Организацией Здравоохранения (ВОЗ) была рекомендована комбинированная терапия, с использованием различных доз препарата артемизинин или его производных (АСТ) , а также иных оральных противомалярийных препаратов для лечения инфекции *Plasmodium falciparum.* Малый процент рецидивов, уменьшение количества гаметоцитов, значительный рост коэффициента выздоровления. Комбинированная терапия эффективно увеличивала срок возникновения резистентности бактерии-возбудителя к лекарственным препаратам. Выпущенное руководство «Правила и протоколы применения противомалярийных препаратов в Китае» предназначено для обеспечения рационального применения и стандартизации мер лечения малярии в этой стране. Этим руководством была одобрена терапия (АСТ), так как артемизинин является рекомендованным препарат первого ряда для лечения малярии в мире. В ответ на международную инициативу уничтожения малярии, в рамках «Целей развития тысячелетия (ЦРТ)», китайское правительство поставило своей целью полностью избавиться от малярии в 2020 году.

Translated from English version into Russian by Irina Zayonchkovskaya, through

TRANSLATORS WITHOUT BORDERS

Revisión histórica del desarrollo de medicamentos contra la malaria y su aplicación en China

Chang Chen

Sinopsis

En esta revisión se trata el desarrollo a lo largo de los años de los medicamentos contra la malaria en China, entre los que se incluyen la artemisinina, el artemetero, el artesunato y la dihidroartemisinina, así como otros medicamentos sintéticos como la piperaquina, la pironaridina, el benflumetol (lumefantrina) y la naftoquina. En especial, se discuten los efectos curativos de estos medicamentos en el tratamiento de la malaria *falciparum*, incluidos los casos resistentes a la cloroquina. Siguiendo el tratamiento combinado basado en artemisinina (ACT) recomendado por la Organización Mundial de la Salud (OMS), se han utilizado diferentes combinaciones de artemisinina o de su derivado y otros medicamentos antimalaria para el tratamiento por vía oral de infecciones por *Plasmodium falciparum*. Los resultados arrojan un índice de agravamiento bajo, una disminución de los portadores de gametocitos y un ascenso notable de la tasa de curación. El tratamiento combinado también es eficaz contra el desarrollo de resistencia al medicamento en el parásito. A fin de regular y estandarizar la aplicación racional del tratamiento en dicho país, se emitió la normativa "Directrices y régimen de tratamiento para el uso de medicamentos contra la malaria en China", que adopta el ACT como primer medicamento recomendado para el tratamiento de la malaria *falciparum*. En respuesta a la iniciativa global de erradicación de la malaria propuesta por las Naciones Unidas en sus Objetivos de Desarrollo del Milenio, el Gobierno chino tiene como objetivo erradicar la malaria en el país para el año 2020.

Translated from English version into Spanish by Marta Callava Linares, through

TRANSLATORS WITHOUT BORDERS
